# The Gambia National Eye Health Survey 2019: survey protocol

**DOI:** 10.12688/wellcomeopenres.16531.2

**Published:** 2021-10-14

**Authors:** Abba Hydara, Andrew Bastawrous, Suzannah Bell, Dorothy Boggs, Tess Bright, Hannaa Bobat, Julian Eaton, Hannah Faal, Modou Jobe, Min J. Kim, Ben Kirkpatrick, Ian McCormick, John Atta Okoh, Segun Isaac Olaniyan, Andrew M. Prentice, Jacqueline Ramke, Ruth Taylor, Matthew Burton, Islay Mactaggart

**Affiliations:** 1 Sheikh Zayed Regional Eye Care Centre, Kanifing, The Gambia; 2 International Centre for Eye Health, London School of Hygiene & Tropical Medicine, London, UK; 3 Moorfields Eye Hospital NHS Foundation Trust, London, UK; 4 International Centre for Evidence in Disability, London School of Hygiene & Tropical Medicine, London, UK; 5 St Mary's Hospital, Newport, UK; 6 Centre for Global Mental Health, London School of Hygiene & Tropical Medicine, London, UK; 7 CBM Global, Cambridge, UK; 8 University of Calabar Teaching Hospital, Calabar, Nigeria; 9 Medical Research Unit The Gambia, London School of Hygiene & Tropical Medicine, Kanifing, The Gambia; 10 School of Optometry and Vision Science, University of Auckland, Auckland, New Zealand; 11 East London NHS Foundation Trust, London, UK

**Keywords:** Eye health survey, vision impairment, blindness, comorbidity, non-communicable diseases, mental health, disability, assistive technology, mobile tools

## Abstract

Two national surveys of vision impairment and blindness were undertaken in The Gambia in 1986 and 1996. These provided data for the inception of The Gambia’s National Eye Health Programme (NEHP) within the Ministry of Health and Social Welfare. There have been important developments in the eye health services provided by the NEHP in the last 20 years. At the same time, the population has also undergone major demographic changes that may have led to substantial changes in the burden of eye disease.

We conducted a National Eye Health Survey of vision impairment, blindness and its comorbidities in adults in The Gambia in 2019. We examined a nationally representative population-based sample of adults 35 years and above to permit direct comparison with the data available from the previous surveys.

Alongside a comprehensive vision and eye examination, the survey provides nationally representative data on important comorbidities in this population: diabetes, hypertension, obesity, hearing impairment, disability and mental health. Secondly, it estimates access to assistive technologies and eye health services. Thirdly, it is powered to allow a five-year follow up cohort study to measure the incidence and progression of eye disease.

## Introduction

National surveys of vision impairment (VI) and blindness were undertaken in The Gambia in 1986 and 1996 ^1, 2^. The 1986 survey provided baseline data on the prevalence and causes of VI and blindness to support the inception of The Gambia’s National Eye Health Programme (NEHP) within the Ministry of Health and Social Welfare. The 1996 survey was completed on an independent sample using the same sampling and examination techniques to provide updated prevalence estimates and relative risk ratios compared to 1986.

The national all-age prevalence of blindness (presenting visual acuity [VA]<3/60, in the better seeing eye) was 0.7% in 1986 and 0.4% in 1996 (confidence intervals [CI] not reported) ^1, 2^. The age-standardised difference between the estimates was not significant at the national level, but there was a higher relative risk of blindness in 1986 compared to 1996 (age adjusted risk ratio [adjRR] 2.2, 95% CI 1.2 – 3.8%) in the Western Region, where NEHP had first been instigated. Both surveys categorised “low vision” as VA <6/18 and ≥3/60, and a modest increase in this category from 1.4% to 1.6% was observed nationally over the same period (adjRR 0.7, 0.6 – 0.9) ^2^. Data on the prevalence of eye disease highlighted cataract, aphakia, uncorrected refractive errors and corneal infections as the leading causes of blindness and low vision in both studies ^1, 2^.

The 1996 survey also provided an opportunity to investigate the burden of non-communicable diseases (NCDs) in The Gambia. The nationwide prevalence of being overweight and obese were 8.1% and 2.1% respectively, hypertension was 24.2% and diabetes mellitus was 0.3% ^3^.

In the more than twenty years since the last comprehensive eye health survey in The Gambia, the NEHP has developed further. This has included the establishment of a new Regional Eye Care Centre in 2007 and several additional centres offering cataract surgery, distributed across the country. In addition, there has been major investment in the development of refractive error services and new in-country capacity to manufacture spectacles ^4^.

During this same period The Gambia has undergone major demographic changes. The population has grown: from 800,000 in 1986 to 1,170,000 in 1996 and 2,300,000 in 2018 ^1, 2, 5^. Life expectancy has increased from 44 years in 1983 to 62 years in 2018, driving a relative and absolute increase in the proportion of the population who are older and in whom prevalence of VI and blindness is highest ^6, 7^. There has also been considerable migration from rural to urban areas, with an associated change in lifestyle. Globally, increased urbanisation has been linked to increases in the prevalence of NCDs, particularly diabetes and hypertension ^8^. Taken together, it is likely that the current population burden of eye disease in The Gambia differs substantially from previous estimates. To address this need for updated eye health data, we conducted a national survey of eye health and its comorbidities between February and July 2019.

Comprehensive eye health surveys are relatively resource intensive in comparison to commonly used rapid methodologies, such as the Rapid Assessment of Avoidable Blindness (RAAB) ^9^. RAAB uses simplified examination procedures and equipment and only samples the population 50 years and older (blindness prevalence is higher in this group than among all ages) ^10^. RAAB provides a substantial proportion of Global Burden of Disease data on vision impairment and blindness ^11^, but recent data comparing RAAB outputs to a more comprehensive methodology are lacking. As an additional objective, we nested the RAAB methodology within this comprehensive survey methodology, to compare findings from a comprehensive versus rapid methodology on the same sample.

This protocol has been prepared to provide a detailed summary of the survey methods, sample characteristics and analytical approaches, in advance of results to be published later in 2021.

## Protocol

### Study aim

To assess the prevalence of vision impairment and its causes and comorbidities in a nationally representative population-based sample of adults 35 years and older in The Gambia, and compare this with the situation in 1996.

### Study objectives

1. To estimate the prevalence and causes of vision impairment and blindness in The Gambia in adults 35 years and older, and in the sub-group 50 years and older, stratified by sex
2. To estimate the prevalence of cataract, corneal blindness/ocular trauma, uncorrected refractive error, trichiasis, glaucoma, diabetic retinopathy and age-related macular degeneration in the Gambia in adults 35 years and older and 50 years and older
3. To evaluate the impact of current Gambia National Eye Health Programme activities, including the provision of cataract and refractive error services
4. To estimate the prevalence of diabetes, hypertension and associated risk factors (body mass index, alcohol and tobacco) of NCDs in the Gambia in adults 35 years and older, and relate these to ocular health
5. To estimate the prevalence of hearing impairment, musculoskeletal impairment, disability and mental health limitations in the Gambia in adults 35 years and older, and relate these to ocular health and the need for vision and hearing assistive products
6. To establish a phenotyped baseline for a long-term eye health cohort study
7. To compare outputs from a comprehensive eye health survey to a rapid methodology

### Sample frame and size

**Figure 1.**
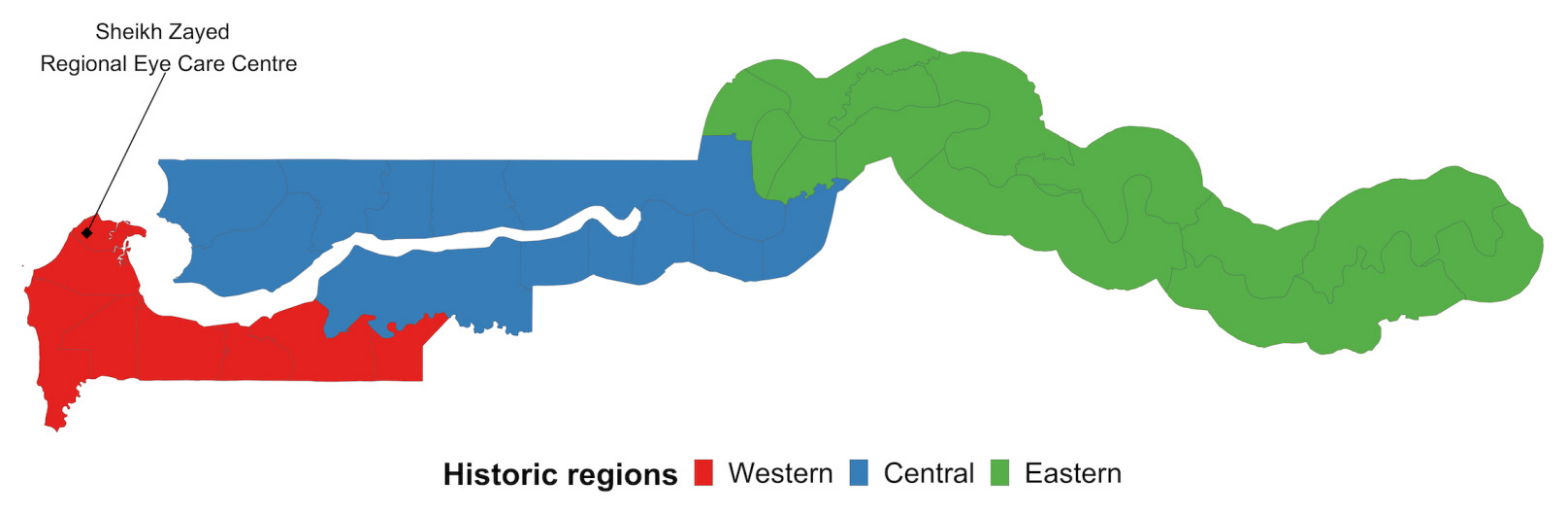
Historic regions of the Gambia.

The 2013 Gambia National Census population estimates were used as the sampling frame ^12^. Multi-stage stratified cluster random sampling with probability proportional to size procedures were used to identify a nationally representative sample of adults 35 years and older, in clusters of 30. Clusters of 30 were selected as the pragmatic number of examinations each team could complete per day. These were selected from standard Gambia Bureau of Statistics (GBoS) Census Enumeration Areas (EAs). The country was divided into three broad regions for comparability to the 1996 estimates: Central, Eastern and Western ( Figure 1). Each of these regions was further stratified to reflect urban and rural population proportions, using Gambia Bureau of Statistics’ definitions.

The sample was powered to detect disease prevalence as low as 0.5% based on relevant literature on glaucoma, diabetic retinopathy and blindness prevalence in the region ^2, 13, 14^. The calculation included a design effect of 2.5 to account for cluster sampling, assuming that samples would be moderately clustered, with an intraclass correlation coefficient (ICC) of 0.038 in clusters of approximately 30 adults 35 years and older ^15^. Accounting for response/follow-up drop-out rate of 20%, regional and urban/rural stratification, and stratification by 35 years and older and 50 years and older, the 5-year expected incidence rate of blindness, and a binomial exact distribution with an estimated margin of error of 0.25% to account for rare conditions (p<0.1), the overall sample size calculated was 10,800 adults age 35 years and older in 360 clusters of approximately 30 adults per cluster.

### Team composition and training

Four teams collected the survey data. Each team was comprised of one ophthalmologist, one optometrist or optometry technician, one senior ophthalmic medical assistant (SOMA), one general nurse, one mental health nurse, and two enumerators. There is only one practicing audiology nurse in The Gambia, who joined one of the teams. This was sufficient given an expected prevalence of hearing impairment of 9%, requiring a sample size of 2,700 (1/4 the overall sample) ^16^.

Teams underwent ten days of training in February 2019, including standardised tests of protocol adherence, practice examinations and pilot testing. Questionnaires were pre-tested, and revised where necessary following the pilot. A formal interobserver variability test was completed for vision testing. Compared to an arbitrarily selected gold standard, two teams achieved substantial agreement (0.7 and 0.8, both p<0.001), while one achieved fair agreement (0.4, p<0.001), requiring further consolidation of research protocol material before beginning data collection.

Team ophthalmologists were trained in the conduction of eye examinations according to protocol by the study PI, a senior consultant ophthalmologist. Only two ophthalmologists were available for the entire duration of data collection. Two teams therefore included a number of different ophthalmologists over the course of the data collection, each trained by a predecessor during a minimum two-day handover. The study PI continued to observe the teams regularly throughout data collection, to ensure that protocol was being followed.

### Pre-data collection preparation

Data collection was scheduled to progress from the east to the west of the country, with all four teams travelling together and completing nearby clusters before moving to the next location. An advance team of enumerators moved ahead of survey teams to notify regional administrative stakeholders, sensitise communities (both for cooperation and acceptance) and manage survey logistics. A vehicle maintenance and servicing schedule was prepared and regional fuel suppliers were identified. The Ministry of Health provided five 4-wheel drive vehicles for the study fieldwork, and released 24 clinical and 19 support staff from their roles, to participate in the survey. The Statistician General of GBoS released eight experienced survey field enumerators and a supervisor, and provided the study teams with EA and regional maps.

### Data collection procedures

***Participant recruitment and informed consent.*** Enumerators used EA maps to visit each cluster in advance, complete a household listing of all eligible residents and identify a central location for the examination. At each household, the purpose of the survey was explained verbally to the household head or an adult key informant using a pre-written study participant information sheet ( *Extended data* ^17^).

If the household head or adult key informant agreed to participate, the enumerator recorded the age, sex and relationship to household head of all eligible household members, irrespective of availability.

Household members were eligible if they were 35 or older, residing in a household in the EA and:

- Had lived in the house at least 6 months of the last year
- Ate shared meals with other household members
- Did not pay, and were not paid by, other household members

Once the listing was completed, enumerators segmented the list into groups of 30 participants, numbered these and selected one segment at random by drawing a number out of a hat. Enumerators returned to the selected segment to provide further information to household members about the details of examination at a central location (within the EA) the following day, and to collect a Global Positioning System (GPS) point reading and data on household characteristics and indicators of socio-economic position (see below). Participants were given urine receptacles to fill the following morning and requested not to have breakfast until after the survey team had arrived.

Enrolment was completed the morning after enumeration, when enumerators returned to the household with the team’s general nurse. Written informed consent was collected by fingerprint or signature for each available participant. Eligible participants who were not available after two repeat visits to the household were recorded as non-responders.

***Data collection at the household.*** On the day of the examination, an enumerator and a general nurse first visited each household in the segment. Each participant was provided with a cardboard participant ID slip recording the household data collection outputs. This was used to track completion of each subsequent component of the examination protocol.

Participants first undertook a fasting Boehringer Mannheim glucose test at their household, completed by the general nurse using sterile lancets, test strips and a glucometer (Accu-chek Aviva Meter). If the participant had not fasted (defined as only ingesting water in the last eight hours), the test was recorded as random. Our original protocol also included HbA _1c_ testing using a portable HbA _1c_ machine (A1CNow+, Bayer) and finger blood sample for participants with fasting blood glucose >= 5.6mmol/L, random blood glucose >= 7.8 mmol/L, or a known history of diabetes. However, the ambient field work conditions (temperature and humidity) were such that the HbA _1c_ test performance was unreliable, and consequently this was abandoned.

Urinalysis was completed using Multistix 10 SF Urinalysis strips (Siemens). Tests for leucocytes, nitrates, proteins, blood, glucose, ketones and pH level were recorded on the participant slip. Participants were then invited to receive breakfast or lunch (staggered per 10 participants to avoid congestion at field stations) at the central location prior to the remainder of the survey assessment.

***Data collection at the central location.*** Participant attendance was recorded on entry at the central location, and data collected at the household was transferred from the participant ID slip to a mobile data collection form on a Huawei MediaPad M3 tablet device. Assessments were split across several stations within the central location. The participant ID slip was used by team members to document assessment completion and relay information on referrals (see below). The full study questionnaire is available as *Extended data* ^17^.

Demographics and general health assessment

The team general and mental health nurses completed the demographics and general health assessment.

*Demographics and self-reported socio-economic position*

A face photograph was taken of each participant to aid follow up, and demographic data including education, ethnic group and household composition was captured. EquityTool, an objective tool comprised of 12 country-specific assets, was used to generate a relative wealth index ^18^. Three self-reported socio-economic position tools were also used: perceived adequacy of household food consumption, perceived adequacy of household income and a socio-economic ladder question ^19^.

*Anthropometry*

Height was measured using a Leicester height measure Mk II, with participant head positioned in the Frankfurt plane. Weight and body fat percentage were measured using a Tanita BC-545n body composition monitor.

*Blood pressure*

Blood pressure was measured in triplicate, once per arm and then repeated in the arm with the higher reading. The participant was seated, with their arm supported at the level of the heart and resting on a surface, and measured using an automated OMRON-Healthcare 10 Series blood pressure monitor (Omron). Measurements were taken five minutes apart, and an average of the last two measures was recorded for analysis.

*Genetic sample*

A genetic sample was taken for each consenting participant, for archiving and future genetic testing. One upper cheek buccal swab sample was collected per participant using a cyto-brush. Each specimen was sealed in an envelope labelled with the participant ID and stored at room-temperature.

*Self-reported NCD history and risk factors*

Participants responded to a pre-coded questionnaire module on personal and family history of diabetes, hypertension and cholesterol level. Smoking and alcohol consumption habits were recorded and body image and attractiveness were assessed using the Figure Rating Scale ^20^. Medication and treatment history were recorded for known diabetics and hypertensives.

Eye health assessment

Visual acuity was measured indoors by the team optometrist or optometry technician, with no direct sunlight or glare in the direction of the participant or the VA test chart. The vision testing protocol is summarised diagrammatically in *Extended data* ^17^.

*Distance visual acuity:* Monocular distance visual acuity (uncorrected and wearing available correction) was measured using Peek Acuity – a validated Android-deployed ‘tumbling E’ visual acuity test – on the tablet devices ^21^. All participants whose uncorrected (or corrected, if wearing spectacles) visual acuity was less than 6/12 in either eye underwent 1) a pinhole test in the eye(s) less than 6/12 (Lorgnette multi 17 occluder) and 2) objective and subjective refraction of both eyes using a trial lens set and fixed wall chart (3 metre Snellen chart, Sussex Vision). Monocular best corrected visual acuity (BCVA) was measured with Peek Acuity following refraction.

*Near vision screening:* Binocular near vision screening was carried out with participants wearing near correction, if available (i.e. presenting near vision). A binary outcome of can (at least 4/5 optotypes correct), or cannot, read an N8 crowded tumbling E optotype at 40cm was recorded. If participants could not see N8 with presenting near vision they were corrected with age-appropriate near addition lenses in a trial frame and retested at the same threshold.

*Contrast sensitivity:* Monocular and binocular contrast sensitivity was measured using the smartphone-based Peek Contrast test deployed on a Sony Z3 smartphone ^22^. The test presented successively lower contrast tumbling E optotypes until they were no longer distinguishable from the background. The test provided a contrast sensitivity measure calibrated to the Pelli-Robson contrast sensitivity test, and an average measure of the ambient light in lux.

*Intraocular pressure:* Intraocular pressure (IOP) was measured by the team’s SOMA using an iCare ic100 tonometer according to device specifications. Time of testing was recorded, and the first eye measured was alternated between participants to avoid operator bias. Unless contra-indicated by current corneal infection, each iCare probe tip was disinfected and used six times before disposal ^23^.

*Ocular examination and imaging:* The team’s ophthalmologist examined both eyes. First, the standard RAAB examination procedure was completed. This included undilated direct ophthalmoscopy examination of the anterior segment and fundus and a lens status screen with pen torch. The RAAB algorithm, whereby the most readily treatable condition only is recorded, was applied to categorise the main cause of VI (presenting <6/12) per eye and per person ^9^. This was undertaken to allow the RAAB methodology-derived diagnosis of cause of VI to be compared with the findings of the subsequent detailed and dilated examination.

The eyelids and anterior segment of the eye (conjunctiva, sclera, cornea, iris and lens) were then examined in detail using a slit-lamp, to document presence of anterior segment eye disease and trachomatous trichiasis using a standardised eye health survey examination form comparable to the 1996 survey methodology. Table 1 describes the study’s outcome measures, including where specific, published grading protocols for classifying particular eye diseases were followed.

A complete assessment at the central location took roughly 1.5 hours, but varied depending on the participants’ health status and according to how many other participants were attending the central location at the time.

**Table 1.** Definitions for the study’s primary and secondary outcome measures.

Primary Outcome Measures		
Measure	Category	Definition
Distance Vision Impairment	Any Vision Impairment	Presenting distance visual acuity (PVA, with available correction if worn) <6/12 in the better seeing eye
	No Vision Impairment	PVA ≥ 6/12 in the better seeing eye
	Mild Vision Impairment	PVA <6/12 and ≥ 6/18 in the better seeing eye
	Moderate Vision Impairment	PVA <6/18 and ≥6/60 in the better seeing eye
	Severe Vision Impairment	PVA <6/60 and ≥3/60 in the better seeing eye
	Blind	PVA <3/60 in the better seeing eye
Sub-categories of blindness	Not blind	PVA ≥ 3/60in the better seeing eye
	<3/60 – 1/60	PVA <3/60 and ≥ 1/60 in the better seeing eye
	<1/60 – Light Perception	PVA ≥ 1/60 and light perception in the better seeing eye
	No Light Perception	No light perception in the better seeing eye
Low Vision (1996 paper comparison)	Low Vision	PVA <6/18 and ≥3/60 in the better seeing eye
Near Vision Impairment	Presenting Near Vision Impairment	Cannot see N8 (binocular), with available correction if worn
	Corrected Near Vision Impairment	Cannot see N8 (binocular), whilst wearing near correction
***Secondary Outcome Measures (ocular, per eye)***		
**Anterior Segment Eye Disease**	Any Refractive Error	Uncorrected visual acuity (UCVA) <6/12 improving to ≥ 6/12 with available correction, pinhole or refraction
	Vision Impairing Refractive Error	Presenting visual acuity (PVA) <6/12 improving to ≥ 6/12 with pinhole or refraction
	Cataract ^24^	Any grade 1 - 3 of nuclear, cortical or posterior capsular cataract or, if ungradable, any cataract marked mature or hypermature using WHO Cataract Grading Tool
	Cataract Surgical Complications	Aphakia, posterior capsular opacification, aphakic bullous or pseudophakic bullous keratopathy identified on ophthalmic examination
	Trachoma corneal opacity ^25, 26^	Current trichiasis (defined using WHO 2019 definition), or evidence of prior trichiasis surgery alongside corneal scarring (C2a – C4 only) in the same eye
	Other corneal opacity ^26^	Corneal scarring but no prior trichiasis or prior trichiasis surgery in the same eye (C2a – C4 only)
	Other anterior segment eye disease	Presence of at least one of the below pre-coded diseases, identified on slit lamp examination: pterygium (cornea involved), band keratopathy, corneal ulcer, uveitis, or other anterior segment ocular disease *or* other anterior segment disease described in open text
**Posterior Segment Eye Disease**	Age-related maculopathy and degeneration (ARMD)	Any ARMD including: drusen or hypo/hyper pigmentation without degeneration, dry or geographic, or wet/neovascular or disciform
	Glaucoma ^27^	99.5% percentile of cup-disc ratio or asymmetry (Category 2), based on field grading. If optic disc not visible: PVA <3/60 and IOP in the 99.5% percentile
	Any diabetic retinopathy ^28^	Any diabetic retinopathy at least R1 or M1 using the Scottish Grading System, based on dilated ocular photograph grading
	Sight-threatening diabetic retinopathy (STDR) ^29^	Proliferative Retinopathy (R4) or Referable Maculopathy (M2) using the Scottish Grading System, based on dilated ocular photograph grading
	Optic disc atrophy	Optic disc atrophy marked as present but does not meet glaucoma definition
	Other posterior segment eye disease	Presence of pseudo-exfoliation, identified on slit lamp examination *or* other posterior segment disease described in open text
	Main cause of distance vision impairment	In all eyes with PVA<6/12, disease presence as above. If more than one of the above definitions are met in one eye using the definitions above, the main cause will be listed as the highest ranking in order of: 1. Refractive Error 2. Cataract 3. Other Anterior Segment 4. Posterior segment 5. Globe 6. Unknown If more than one of the above definitions is met in one person, the main cause at the person level will be listed as the highest ranking in this order. Participants with PVA<6/12 with no reported anterior or posterior segment disease as defined above were categorised as unknown. A known limitation of this hierarchical approach to determining the “main cause” is that it will lead to under estimation of posterior segment causes. The proportion of people with comorbidities will be reported, and manuscripts detailing prevalence and associations of specific eye diseases will provide further detailed breakdown on anterior and posterior causes of VI.
Service Coverage	Cataract Surgical Coverage (CSC)	Proportion of people with operated cataract (pseudophakia/aphakia) as a proportion of all people with operated cataract or operable cataract (defined at different thresholds of BCVA)
	Refractive Error Coverage (REC)	Proportion of people with refractive error (UCVA<6/12 in the better eye, correctable to 6/12 or better) with refractive error correction
Effective Service Coverage	Effective Cataract Surgical Coverage (eCSC) ^30^	Proportion of people with operated cataract (pseudophakia/aphakia) and good postoperative presenting visual acuity (VA 6/12 or better) as a proportion of all people with operated cataract or operable cataract (defined at different thresholds of BCVA)
	Effective Refractive Error Coverage (eREC) ^31^	Proportion of people with refractive error (UCVA<6/12 in the better eye, correctable to 6/12 or better) with refractive error correction and a good outcome (CVA 6/12 or better)
***Secondary Outcome Measures (non-ocular)***		
Hypertension		Average systolic blood pressure values across two readings of ≥140 mmHg and/or diastolic values of ≥90 mmHg and/or taking antihypertensive medication and/or reported history of hypertension
Diabetes	Diabetic	Reported history of diabetes (told by healthcare worker and/or on diabetic treatment), fasting blood glucose (FBG) ≥7mmol/L or random blood glucose (RGB) ≥11mmol/L
	Pre-diabetic	FBG >5.6 <7, or RBG ≥7.8 <11
	Not diabetic	No reported history of diabetes and neither impaired FBG or RBG
Obesity	Underweight	Body Mass Index (BMI) under 18
	Normal	BMI ≥18 and <25
	Overweight	BMI ≥25 and >30
	Obese	BMI ≥30
Hearing Impairment ^32^	None	>19 decibels hearing level (dbHL) jn either ear
	Mild	20 to <35 dBHL in the better hearing ear
	Moderate	35 to <50 dBHL in the better hearing ear
	Moderately Severe	50 to <65 dBHL in the better hearing ear
	Severe	65 to <0 dBHL in the better hearing ear
	Profound	80 to <95 dBHL in the better hearing ear
	Complete/ total	95 dBHL or greater in the better hearing ear
	Binary Classification	20dbHL or greater in the better hearing ear
Anxiety ^33^	None	Score of 0-4 on GAD-7
	Mild	Score of 5-9 on GAD-7
	Moderate	Score of 10-14 on GAD-7
	Severe	Score of ≥15- on GAD-7
	Binary Classification	Score of ≥ 10 on GAD-7
Depression ^34^	None	Score of 0-4 on PHQ-9
	Mild	Score of 5-9 on PHQ-9
	Moderate	Score of 10-14 on PHQ-9
	Moderately Severe	Score of 15-19 on PHQ-9
	Severe	Score of 20-27 on PHQ-9
	Binary Classification	Score of ≥ 10 on PHQ-9
Disability		Any of the 6 Washington Group Short Set Functional Domains reported “a lot of difficulty” or “cannot do”

Unless contra-indicated (IOP ≥35mmHg or van Herrick’s grade 2 or 1 was recorded), all participants were then dilated in both eyes using the short-acting mydriatic eye drop tropicamide 1%. A slit lamp and a 90D fundus lens, were used to complete a comprehensive examination and grade predetermined lens, retinal and optic disc disease.

Imaging was completed by the team’s SOMA. The anterior segment of both eyes was photographed using a Nikon D5600 Digital Single Lens Reflex (SLR) camera with macro lens and flash. The posterior segment was photographed (disc centred and macula centred images) using the Remidio Retinal Camera imaging system ^35^.

Other impairment and functioning assessment

*Self-reported functioning:* The team general nurse used the Washington Group Short Set to measure self-reported functional limitations in seeing, hearing, walking/climbing, remembering/concentrating, understanding/being understood and selfcare ^36^. Mental Health was assessed by the mental health nurse using two well-established tools: The Patient Health Questionnaire 9 (PHQ 9) for measuring depression ^37^, and the Generalised Anxiety Disorder 7 item tool (GAD-7) ^38^, for anxiety.

*Self-reported assistive product use and need:* The general nurse asked reported need for, use of and barriers to access to assisted products (including glasses) using a modified version of the World Health Organisation (WHO) rapid assistive technology assessment (rATA) ^39^.

*Musculoskeletal impairment:* The general nurse used the six screening questions from the Rapid Assessment of Musculoskeletal impairment to screen for musculoskeletal impairment (MSI) ^40^.

*Hearing impairment:* In the team measuring hearing impairment, an audiology nurse screened for hearing impairment using HearTest, a validated mobile pure tone audiometry application deployed on a Samsung Galaxy A3 Smartphone together with calibrated, noise-cancelling Sennheiser HD280 pro circumaural headphones ^41^. Hearing tests were completed in a separate and private area, and ambient noise levels were automatically recorded by the device, which flagged a warning when these reached unacceptable levels. Following the Rapid Assessment of Hearing Loss (RAHL) methodology, all participants screened for hearing impairment also had their ears briefly examined by the team audiology nurse to assess ear health, and if applicable determine cause of hearing loss and appropriate referral mechanisms ^16^.

### Diagnoses and referrals

Survey teams carried basic first aid kits and medicines for treating common illnesses, and referral letters for onward services. Referrals for eye conditions were made to the Sheikh Zayed Regional Eye Care Centre in Kanifing, close to the capital city Banjul. Participants with blood pressure readings above 95 mm/Hg diastolic or 150 mm/Hg systolic, alongside participants judged by the team general nurse to require follow up services for other reasons (including emergencies) were referred to relevant primary health services. The team mental health nurses made referrals to relevant mental health services as per their clinical judgement following screening. Any participant with hearing impairment ≥35 dBA in the better ear or who was otherwise considered in need of referral by the audiology nurse was referred to the relevant ENT services.

### Data management

Data collection forms were built using Open Data Kit (ODK) software ^42^. Tablets were password protected and team leaders used data SIM cards to transfer the encrypted data to a secure ODK server held at the London School of Hygiene & Tropical Medicine (LSHTM) daily. Electronic data support was provided by LSHTM Global Health Analytics (odk.lshtm.ac.uk). During data collection, anterior segment images were stored locally on password-protected laptop computers and backed up weekly to password-protected storage drives. After data collection, all images were transferred to a secured LSHTM server.

Anonymised posterior segment images were transferred via WiFi daily to a secured cloud-based platform. Fundus image grading for diabetes, AMD and glaucoma will be performed remotely by trained ophthalmologists, following a formal training and inter-observer variation test.

### Data preparation

Data collection was completed between March and July 2019. Raw data were exported from the secure server and imported into STATA version 14.0. Data were merged into a single database and anonymised.

**Figure 2.**
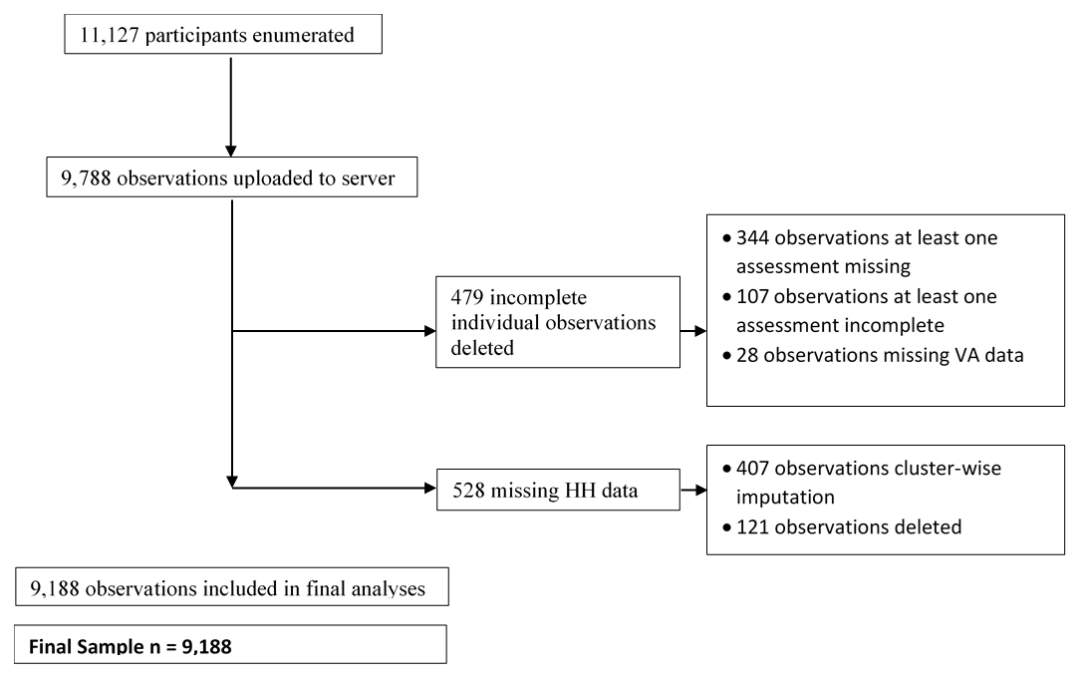
Flow chart of data completeness.

***Data completeness***. To prevent listwise deletion, all data were checked for completeness. Figure 2 summarises this process.

***Sample characteristics.*** Table 2 presents the final sample population characteristics, compared with the characteristics of the population in the 2013 Census ^12^. The survey oversampled women compared to men (70.3% female vs. 29.7% male). Additionally, selection probabilities were lower than expected in several age groups (5-year band) and in clusters.

**Table 2.** Sample characteristics.

	Sample, n (%)	Census 2013, n (%)
**Age Group**		
35 – 44	4,102 (44.7)	167,595 (43.7)
45 – 54	2,061 (22.4)	101,183 (26.4)
55 – 64	1,444 (15.7)	56,894 (14.8)
65 – 74	1,018(11.1)	33,755 (8.8)
75 – 84	441 (4.8)	16,521 (4.3)
85+	122 (1.3)	7779 (2.0)
Mean (SD)	49.6 (13.4)	
**Sex**		
Male	2,710 (29.5)	192,969 (50.3)
Female	6,478 (70.5)	190,758 (49.7)
**Region**		
Central	1,476 (16.1)	301,122 (16.2)
East	2,087 (22.7)	459,127 (24.7)
West	5,625 (61.2)	1,096,932 (59.1)
**Location**		
Rural	4,149 (45.2)	783,884 (42.2)
Urban	5,039 (54.8)	1,073,297 (57.8)
**Ethnicity**		
Mandinka/Jahanka	3,564 (38.8)	120,000 (34.9%)
Wollof	1,365 (14.9)	50,494 (14.7%)
Jola/Karoninka	1,079 (11.7)	41,820 (12.1%)
Fula/Tukulur/ Lorobo	1,847 (20.1)	76,753 (22.3%)
Serere	287 (3.1)	11,570 (3.4%)
Serahuleh	677 (7.4)	25,442 (7.4%)
Creole and AkuMarabo	22 (0.2)	2,570 (0.7%)
Manjago	171 (1.9)	7,095 (2.1%)
Bambara	69 (0.8)	3,822 (1.1%)
Other ethnic group	103 (1.1)	4,653 (1.3%)
Non-Gambian	4 (0.0)	
**Socio-economic** **position (SEP)** **quintile**		
1 ^st^ (Poorest)	853 (9.3)	
2 ^nd^	1,313 (14.3)	
3 ^rd^	2,251 (24.5)	
4 ^th^	2,121 (23.1)	
5 ^th^ (Richest)	2,650 (28.8)	

Poststratification sample weights were calculated to account for the disproportionate age-sex sampling by 5-year band. Two sample weights were created, one to generalize the findings to the 2013 Gambia Census ^12^, and one to the WHO Standard Population ^43^. All weights were then multiplied with the cluster selection probabilities.

***Defining outcome measures.*** Table 1 describes the definitions for the study’s primary and secondary outcome measures.

***Socio-economic position imputation.*** Quintiles based on the Gambia Demographic and Health Survey 2013 were established following EquityTool procedures. To improve the integrity of socioeconomic position (SEP) data, all 12 EquityTool questions were checked for completeness. Preliminary analysis revealed that among all 360 clusters, 67 had at least one participant with one or more questions unanswered. Missing data were handled by re-approaching non-respondents of 23 clusters where more than half of its participants had incomplete SEP data.

For the remaining observations missing data, mean imputation was used in which the most frequent value of a cluster filled the missing attribute’s value. Each of the EquityTool questions was treated independently of other questions and of other clusters. Missing values were not substituted if there was more than a single most frequent response observed for that attribute.

### Ethics

Ethical approval for the study was granted in 2019 by the Gambia Government/MRC Joint Ethics Scientific Coordinating Committee (SCC, Ref 1635) and the LSHTM Observational/ Interventions Ethics Committee (Ref 16172).

### Dissemination, engagement and data availability

A summary of survey findings will be shared with relevant stakeholders through the Directorate of Planning and Information (DPI) of the Ministry of Health. Study results will be published in a suite of peer-reviewed manuscripts later in 2021 and beyond. The study team includes the National Eye Health Coordinator in the Gambia (AH), ensuring that results will feed directly into population eye health service planning. The anonymised dataset will be made available on reasonable request from the study team.

### Study status

Data has been collected and prepared for analysis. Data analysis is ongoing across different study objective areas.

### Strengths and limitations

The data from the Gambia National Eye Health Survey 2019 will provide valuable, robust data on population eye health and comorbidities in a nationally representative sample of the population of the Gambia 35 years and older. We used validated tools and collected data in line with international priorities and the Universal Health Coverage agenda, and maximised comparability to the previous survey by using similar screening and examination tools. The inclusion of modules on disability, hearing, musculoskeletal impairment, mental health and NCDs will support evidence-based service provision and greater understanding of comorbidities. The phenotyping and sample adjustment to support establishment of a cohort study may provide powerful data on the incidence and progression of disease.

There were also limitations. The comprehensive nature of the protocol led to higher than expected incomplete examinations and non-response rates, requiring sampling weights to be applied. The 2019 survey fieldwork did not include visual fields testing unlike the 1996 survey that used the Henson Visual Fields Analyzer. While we took advantage of newer hand-held techniques where appropriate, it was logistically challenging to set up central locations in each cluster without electricity to power table-top/table-mounted equipment, quiet areas for hearing testing and a food preparation area for participant lunches; all of which contributed to occasional delays for participants. Further, conditions did not allow us to proceed with HbA _1c_ testing, and human resource constraints did not permit continuity of examiners, potentially leading to measurement bias. Two teams had high turnover of ophthalmologists at various stages of the data collection. These human resource challenges meant some clusters had to be revisited in order to examine 80% or more listed participants.

The period April to July in The Gambia coincides with the pre-rainy and rainy/farming season, which sees most rural Gambian men 35 years and older spending more time in their farms. This social pattern skewed the population that was available on the morning of examination towards females, leading to a requirement for poststratification weighting of the sample results in all analyses.

## Conclusion

The Gambia National Eye Health Survey 2019 will provide data to support eye health and broader health service planning in The Gambia and allow critical appraisal of changes in the population’s eye health needs in comparison to earlier national surveys of 1986 and 1996. This survey shall provide a basis to explore the broader understanding of the evolution of chronic and blinding eye diseases and other co-morbid health conditions in a rapidly increasing West African population.

## Data availability

### Underlying data

No data were associated with this article.

### Extended data

Open Science Framework: Gambia National Eye Health Survey 2019 Study Documents, https://doi.org/10.17605/OSF.IO/EKCDT ^17^.

This project contains the following extended data:

- Study questionnaire
- Informed consent sheet
- Vision testing protocol

Data are available under the terms of the Creative Commons Attribution 4.0 International license (CC-BY 4.0).
