# Supplementary material for: The Gambia National Eye Health Survey 2019: survey protocol
Source: Wellcome Open Res. 2021 Oct 14;6:10. Originally published 2021 Jan 21. [Version 2] doi: 10.12688/wellcomeopenres.16531.2 (PMC8591516; doi:10.12688/wellcomeopenres.16531.2)
Supplement: Peer review history

# Peer review: Reviewer response for version 2

**DOI:** 10.21956/wellcomeopenres.19114.r46435

**Published:** 2021-11-11

**Authors:** Ling Lee

**Affiliations:** 1 University of New South Wales, Sydney, NSW, Australia; 2 Murdoch Children's Research Institute, Melbourne, Vic, Australia; 3 University of Melbourne, Melbourne, Vic, Australia

My sincerest apologies for the delay. Thank you for your considerate responses, I have no further comments to make.

Is the study design appropriate for the research question?

Partly

Is the rationale for, and objectives of, the study clearly described?

Yes

Are sufficient details of the methods provided to allow replication by others?

Yes

Are the datasets clearly presented in a useable and accessible format?

Not applicable

Reviewer Expertise:

Health services research and evaluation, Quality of refractive error care, Ocular Epidemiology, Genomic secondary and incidental findings.

I confirm that I have read this submission and believe that I have an appropriate level of expertise to confirm that it is of an acceptable scientific standard.

# Peer review: Reviewer response for version 2

**DOI:** 10.21956/wellcomeopenres.19114.r46436

**Published:** 2021-10-21

**Authors:** Srinivas Marmamula

**Affiliations:** 1 Brien Holden Institute of Optometry and Vision Science, Gullapalli Pratibha Rao International Centre for Advancement of Rural Eye care, L V Prasad Eye Institute, Hyderabad, Telangana, India

Thank you for revising the manuscript based on my comments. Congratulations on this great work. I have no further comments to make.

Is the study design appropriate for the research question?

Yes

Is the rationale for, and objectives of, the study clearly described?

Yes

Are sufficient details of the methods provided to allow replication by others?

Yes

Are the datasets clearly presented in a useable and accessible format?

Not applicable

Reviewer Expertise:

public eye health specialist; optometrist

I confirm that I have read this submission and believe that I have an appropriate level of expertise to confirm that it is of an acceptable scientific standard.

# Peer review: Reviewer response for version 1

**DOI:** 10.21956/wellcomeopenres.18215.r44224

**Published:** 2021-06-17

**Authors:** Srinivas Marmamula

**Affiliations:** 1 Brien Holden Institute of Optometry and Vision Science, Gullapalli Pratibha Rao International Centre for Advancement of Rural Eye care, L V Prasad Eye Institute, Hyderabad, Telangana, India

It is a well-written paper vividly describing the study protocol. I have a few comments to improve the manuscript.

1. Mention of age groups 35 and older and 50 and older are confusing. As the protocol is the same for all age groups, suggest using 35 years and older in the objectives and elsewhere in the manuscript instead of both the age groups.
2. Four teams were used in the survey. On average, how many participants were examined on each day by a single team?
3. How was the quality of eye examinations in the field monitored?
4. What was the time taken for the assessment of each participant in the central location?
5. What was the purpose of the genetic sample?
6. Lower participation of men is a matter of concern and authors should discuss the implications of this on the extrapolation of the results to the population. Also, the reasons for this large difference should be presented though authors describe harvesting season as one reason.

Is the study design appropriate for the research question?

Yes

Is the rationale for, and objectives of, the study clearly described?

Yes

Are sufficient details of the methods provided to allow replication by others?

Yes

Are the datasets clearly presented in a useable and accessible format?

Not applicable

Reviewer Expertise:

public eye health specialist; optometrist

I confirm that I have read this submission and believe that I have an appropriate level of expertise to confirm that it is of an acceptable scientific standard, however I have significant reservations, as outlined above.

# Response

**Published:** 2021-10-08

**Authors:** Islay Mactaggart

**Affiliations:** London School of Hygiene & Tropical Medicine, UK

It is a well-written paper vividly describing the study protocol. I have a few comments to improve the manuscript.

**Response: We thank Dr. Marmamula for their consideration of our manuscript and helpful comments below.**

 Mention of age groups 35 and older and 50 and older are confusing. As the protocol is the same for all age groups, suggest using 35 years and older in the objectives and elsewhere in the manuscript instead of both the age groups.

**Response: Objective 7 is relevant to the population 50+ only. We have revised Objective 1 in the revised manuscript, in hope that this reduces any confusion.**

****

 Four teams were used in the survey. On average, how many participants were examined on each day by a single team?

**Response: Each team completed one cluster of 30 per day. We have described this in the sample frame and size section. Unfortunately we do not have additional data on the average number actually seen per team per day beyond this.**

 How was the quality of eye examinations in the field monitored?

**Response: Team ophthalmologists were trained in the conduction of eye examinations according to protocol by the study PI, a senior consultant ophthalmologist. The study PI continued to observe the teams regularly throughout data collection, to ensure that protocol was being followed. We have clarified as such in the “Team composition and training” section of the revised manuscript.**

 What was the time taken for the assessment of each participant in the central location?

**Response: A complete assessment at the central location took roughly 1.5 hours, but varied depending on the participants’ health status and according to how many other participants were attending the central location at the time. We have included this at the end of the section “Data collection at the central location” in the revised manuscript.**

 What was the purpose of the genetic sample?

**Response: The genetic sample was taken for archiving and future genetic testing. The scope of future testing (including depth and breadth of analyses) will be conditional on further funding. We have clarified as such in the revised manuscript.**

 Lower participation of men is a matter of concern and authors should discuss the implications of this on the extrapolation of the results to the population. Also, the reasons for this large difference should be presented though authors describe harvesting season as one reason.

**Response: We are unable to determine explicitly whether other reasons contributed to the low participation of men in the study, as we did not capture data on this. We believe this to be associated with harvesting season. We describe the post-stratification weighting of the sample to account for this, which supports us in being able to extrapolate from the sample to the population. We have clarified this in the Strengths and Limitations section of the revised manuscript.**

# Peer review: Reviewer response for version 1

**DOI:** 10.21956/wellcomeopenres.18215.r42596

**Published:** 2021-03-01

**Authors:** Ling Lee

**Affiliations:** 1 University of New South Wales, Sydney, NSW, Australia; 2 Murdoch Children's Research Institute, Melbourne, Vic, Australia; 3 University of Melbourne, Melbourne, Vic, Australia

This method article aims to investigate the prevalence of vision impairment, blindness and associated comorbidities in a population-based cohort of adults aged 35 years and older in The Gambia. Overall, it is well written, and largely, a well-considered protocol with all the different components of data collection that draw upon validated tools/measures, yet still remain relatively practical. With a large cohort recruited, there is great opportunity to assess the information with various perspectives, enables at least some comparison to 1996 data, to rapid methodology and provides a good baseline for future national surveys.

 A few areas that would benefit from clarification:

- Why was a genetic sample taken? It does not appear to be associated with any of the objectives. Is it to develop a genotype-phenotype baseline?
- I can see in the Extended data, you mention genetic testing for associated ocular conditions, would this be single gene testing, or would it be wider panels/exome/genome sequencing? Is there a risk of incidental findings? If so, there does not appear to be appropriate information considering the potential ethical implications of genetic testing even for research purposes.
- For study objective 3, it is unclear which outcome measures are used to measure/evaluate the impact of Gambia NEHP activities such as cataract and refractive error services. I wonder whether prevalence of VI or blindness is enough. There does not appear to be the inclusion of cataract surgical outcomes and only presenting distance and near VA with correction might be possible to measure refractive error services (that does not take into account those who purchase readymade spectacles from elsewhere). 
- With newer technology and up to date validated techniques used for the primary and secondary outcome measures, how comparable will these outcomes be to 1996 Survey data?
- Minor edit: Please spell out or provide a footnote for the socioeconomic position in Table 2.

Congratulations on completing the data collection. I look forward to seeing the outcomes from this Survey.

Is the study design appropriate for the research question?

Partly

Is the rationale for, and objectives of, the study clearly described?

Yes

Are sufficient details of the methods provided to allow replication by others?

Yes

Are the datasets clearly presented in a useable and accessible format?

Not applicable

Reviewer Expertise:

Health services research and evaluation, Quality of refractive error care, Ocular Epidemiology, Genomic secondary and incidental findings.

I confirm that I have read this submission and believe that I have an appropriate level of expertise to confirm that it is of an acceptable scientific standard, however I have significant reservations, as outlined above.

# Response

**Published:** 2021-10-08

**Authors:** Islay Mactaggart

**Affiliations:** London School of Hygiene & Tropical Medicine, UK

This method article aims to investigate the prevalence of vision impairment, blindness and associated comorbidities in a population-based cohort of adults aged 35 years and older in The Gambia. Overall, it is well written, and largely, a well-considered protocol with all the different components of data collection that draw upon validated tools/measures, yet still remain relatively practical. With a large cohort recruited, there is great opportunity to assess the information with various perspectives, enables at least some comparison to 1996 data, to rapid methodology and provides a good baseline for future national surveys.

**Response: We thank Dr. Lee very much for this positive and helpful feedback on the manuscript, and respond to their specific comments further below.**

 A few areas that would benefit from clarification:

 Why was a genetic sample taken? It does not appear to be associated with any of the objectives. Is it to develop a genotype-phenotype baseline?

 I can see in the Extended data, you mention genetic testing for associated ocular conditions, would this be single gene testing, or would it be wider panels/exome/genome sequencing? Is there a risk of incidental findings? If so, there does not appear to be appropriate information considering the potential ethical implications of genetic testing even for research purposes.

**Response: The genetic sample was taken for archiving and future genetic testing. The scope of future testing (including depth and breadth of analyses) will be conditional on further funding. We have clarified as such in the revised manuscript.**

 For study objective 3, it is unclear which outcome measures are used to measure/evaluate the impact of Gambia NEHP activities such as cataract and refractive error services. I wonder whether prevalence of VI or blindness is enough. There does not appear to be the inclusion of cataract surgical outcomes and only presenting distance and near VA with correction might be possible to measure refractive error services (that does not take into account those who purchase readymade spectacles from elsewhere). 

**Response: We omitted to describe cataract surgical coverage and refractive error coverage (plus effective [quality-corrected] measures of the above) as outcome measures in Table 1. These have been included in in the revised manuscript and will be explored in the relevant results papers to evaluate the impact of the NEHP on these service outcome indicators.**

 With newer technology and up to date validated techniques used for the primary and secondary outcome measures, how comparable will these outcomes be to 1996 Survey data?

**Response: We used Peek Acuity to test visual acuity, which has been shown to be comparable with Snellen optotypes, as used in the previous study. Indirect ophthalmoscopy and slit lamp examination were included in 1996 as in 2019 and epidemiological definitions remain similar. Combined, we believe these attributes support comparability between the primary outcomes of both surveys. We have clarified this in strengths and limitations in the revised manuscript.**

****

 Minor edit: Please spell out or provide a footnote for the socioeconomic position in Table 2.

**Response: we have made this change**
